# Vaginal Health in Menopausal Women

**DOI:** 10.3390/medicina55100615

**Published:** 2019-09-20

**Authors:** Stefania Alvisi, Giulia Gava, Isabella Orsili, Giulia Giacomelli, Maurizio Baldassarre, Renato Seracchioli, Maria Cristina Meriggiola

**Affiliations:** 1 Gynecology and Physiopathology of Human Reproduction Unit, S. Orsola-Malpighi Hospital, University of Bologna, 40126 Bologna, Italycristina.meriggiola@unibo.it (M.C.M.); 2 Department of Surgical and Medical Sciences, S. Orsola-Malpighi Hospital, University of Bologna, 40126 Bologna, Italy; 3 Center for Applied Biomedical Research (CRBA), University of Bologna, 40126 Bologna, Italy

**Keywords:** vaginal health, menopausal women, vulvovaginal atrophy, genitourinary syndrome

## Abstract

The aim of this review is to provide an overview of genitourinary health in peri- and postmenopause, particularly of vulvovaginal atrophy (VVA), which is part of genitourinary syndrome (GSM). This condition has a high prevalence among post-menopausal women and negatively affects a woman’s quality of life. Epidemiology, signs, symptoms, diagnostic criteria of VVA and target treatments for restoring vaginal health are discussed in light of the most recent literature. Issues related to this condition in menopausal women are under-diagnosed, lack objective diagnostic criteria, and consequently under-treated. Over the years, many treatments have been developed but their long-term effectiveness and safety have yet to be clearly defined. Patients are often dissatisfied and stop treatment, suggesting the need for a more personalized and tailored approach to achieve better compliance and thereby effectiveness. The aim of this paper is to provide an overview of the most recent literature on VVA in order to help the gynecologist in the management of this condition.

## 1. Introduction

The condition of hypoestrogenism related to menopause has a strong negative impact on vaginal and urinary health, often leading to a condition called genitourinary syndrome (GSM), a term introduced by the International Society for the Study of Women’s Sexual Health and the North American Menopause Society in 2014 [1]. GSM is associated with genital signs and symptoms such as dryness, burning, irritation, and sexual symptoms such as discomfort or pain, and impaired sexual function. This condition, previously known as vulvovaginal atrophy (VVA), may also be accompanied by urinary signs and symptoms such as urinary incontinence, dysuria, stranguria, and frequent urinary tract infections [2]. Unlike other menopausal symptoms, VVA is a chronic condition that tends to worsen throughout the years after menopause. It therefore requires prompt and long-term therapy to achieve good results and to avoid the recurrence of symptoms when treatment is stopped.

The aim of this paper is to provide an overview of the most recent literature on VVA that would help to sensitize the clinician toward the diagnosis and treatment of this condition [3,4]. This condition has important consequences in the daily life of post-menopausal women and in their relationships [5,6]. Considering that women spend a third of their life in menopause, it is essential to recognize and treat this syndrome in order to restore the vaginal and vulvar epithelium and ultimately improve quality of life.

**Figure A1:**
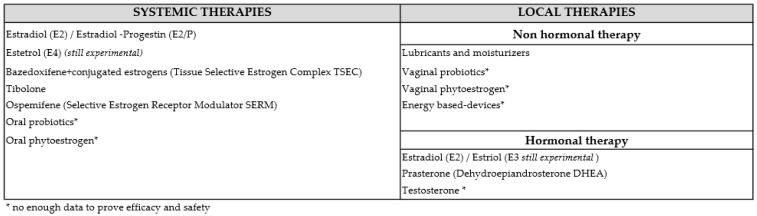
Possible treatment options for vulvovaginal atrophy.

**Figure A2:**
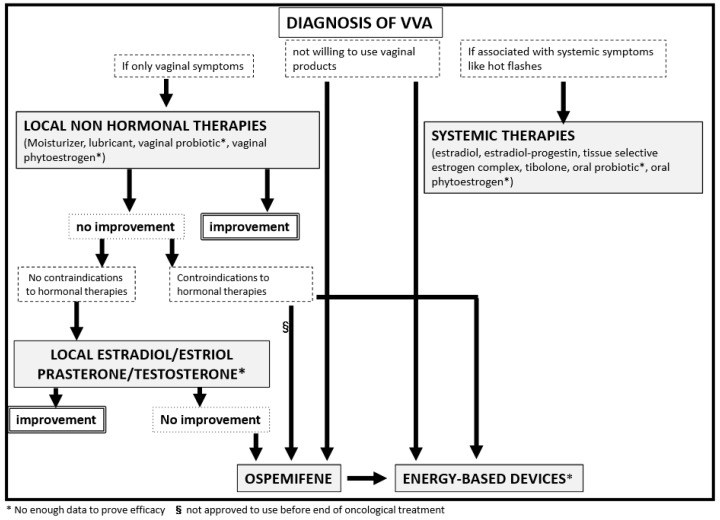
Flow-chart of the management of vulvovaginal atrophy.

The rationale of treatment is the restoration of normal vaginal and vulvar physiology that leads to the alleviation of symptoms. Many options have been developed over the years such as local, systemic hormonal, and non-hormonal treatments or energy-based treatments that could potentially fulfill most women’s needs and preferences, thus improving the quality of post-menopausal women’s lives (Figure A1 and Figure A2).

## 2. Epidemiology of Vulvovaginal Atrophy

VVA affects most peri- and postmenopausal women with a prevalence ranging from 36% to almost 90%, according to the most recent surveys. It has recently been reported that this condition is also already present in pre-menopausal years with a prevalence of 19% in women aged 40–45 (Table A1) [7,8,9,10,11,12,13].

In spite of its high prevalence, VVA is still under-diagnosed and under-treated. Most women do not discuss their symptoms with their gynecologist for various reasons; often because they believe it is just a natural part of aging or because they are uncomfortable talking about it. Often they are unaware that there is treatment for the syndrome, or because of time constrains and/or perceived lack of interest of their healthcare provider. Whatever the reason, the lack of diagnosis still remains one of the major issues in the care of this condition [10,14].

Women tend to self-medicate using over-the-counter drugs that are sometimes ineffective or not effective enough and are therefore stopped, leaving the woman to live with the condition untreated [14].

## 3. Clinical Signs and Symptoms of Vulvovaginal Atrophy

The drop in circulating hormone levels, especially estrogens, represents the main trigger determining vulvovaginal atrophy. The vaginal epithelia of post-menopausal women display flattened epithelial surfaces with features of keratinization and the absence of papillae. Multiple layers of parabasal cells with higher nucleus to cytoplasm ratio and few intermediate and superficial cells are present in which glycogen stores are reduced. This leads to a decrease in the number of Lactobacilli resulting in an increase in vaginal pH [15]. The low percentage of Lactobacilli and the increase in the relative proportion of anaerobic bacteria found in post-menopausal women may predispose symptomatic VVA, although not all studies consistently report this association [16,17,18].

Hypo estrogenic vaginal states typically also include changes in the connective tissue composition with decreased type I/III collagen ratio, which leads to reduced tissue strength [19]. Thinning of the vaginal epithelium increases susceptibility to trauma, resulting in bleeding, petechiae, and ulceration with any type of pressure including sexual activity or a simple gynecological maneuver. Thinning also exposes the underlying connective tissue, which is more vulnerable to inflammation or infection.

Due to these histological changes, clinical signs at the vaginal level include anything from dryness and insufficient hydration, redness, loss of elasticity, petechiae, ulceration, inflammation, atypical secretions, to fibrosis and vaginal obliteration. The most frequent signs at a vulvar level include reduction in tissue thickness, labia agglutination, loss of pubic hair, and scratching lesions due to itching. Consequent symptoms include vaginal dryness and superficial dyspareunia with a prevalence of 78% and 76%, respectively [20], which can be associated with itching, a burning sensation, and susceptibility to mechanical insults, leucorrhoea, or atypical secretions. At a vulvar level, the most frequent symptoms are burning, pain, increased susceptibility to physical and chemical irritants, and mechanical insults [21].

All of these changes have a great impact on women’s sexuality and relationships [22]. The REVIVE study suggested that VVA symptoms have a significant impact on the patients’ ability to achieve pleasurable relations (74%) and spontaneity (70%). Seventy-five percent of sexually active post-menopausal women with VVA were reported to have a significantly reduced sex drive as a direct consequence of the symptoms related to this condition [20]. A 2014 study, showed that most women were worried that vaginal discomfort could have long-term effects on their relationship [23].

## 4. Diagnosis of Vulvovaginal Atrophy

The diagnosis of vulvovaginal atrophy is based on clinical assessment: anamnesis, evaluation of the patient’s symptoms, and gynecological examination with the evaluation of clinical signs. In addition, standardized scores and laboratory tests can be used such as the evaluation of vaginal pH and the vaginal maturation index (VMI). The anamnesis should also include questions about sexual function, the presence of decreased libido, and of dyspareunia. It is important to differentiate superficial dyspareunia, typical of vulvovaginal atrophy, from deep dyspareunia, typical of endometriosis. Moreover, the sexual life of the couple should be investigated from the perspective of the new paradigm of couplepause [24]. Avoiding sexual intercourse can exacerbate VVA as sexual activity can preserve the vaginal epithelium by increasing blood flow and elasticity.

However, there is not always a correlation between clinical signs, laboratory data, and symptoms and this represents an important limitation for diagnosis. Another issue is the subjectivity of the diagnosis. One of the most commonly used scores is the vaginal health index (VHI) [25] for the evaluation of vaginal elasticity, secretions, pH, the presence of petechiae on the epithelial mucosa, and hydration. The score can vary between five and 25, with a cut-off < 15 index of atrophic vagina. The vulvar health index can be used to evaluate the vulva including vulvar inflammation, musculature contraction, pain at speculum insertion, and epithelial integrity. The score can vary from zero to 24, with a cut-off > 8 index of atrophic vulva.

The VMI indicates the degree of tissue maturation, measuring the percentage of superficial, intermediate, and parabasal cells. The maturation value (MV) is calculated with the following formula: MV = % surface cells + (0.5 × % intermediate cells) [26].

A pilot study proposed the use of trans-abdominal ultrasound to measure vaginal wall thickness and total vaginal mucosal thickness at the bladder trigone [27]. Although the study is still preliminary, this could represent a valuable tool for obtaining an objective evaluation of vaginal health and to quantify the response to therapeutic interventions.

## 5. Treatment Options for Vulvovaginal Atrophy

### 5.1. Lubricants and Moisturizers

Various non-hormonal, non-prescription treatments exist for vaginal atrophy (VA), namely increased coital activity, cessation of smoking, pelvic-floor physiotherapy (PT), and lubricants or moisturizers [22]. Many women with VVA use over-the-counter (OTC) products such as vaginal lubricants and moisturizers. International guidelines consider these to be the first line of therapy in the treatment of VVA being free from significant contraindications and side effects [28]. They can be used alone or in combination with hormonal therapies as needed. This treatment option is also recommended for women for whom the use of vaginal estrogen preparations is unacceptable. It is important that osmolality, pH, and the composition of these products, either lubricants or moisturizers, are similar to vaginal secretion [29].

The main difference between vaginal lubricants and moisturizers is the timing of application. Vaginal lubricants are particularly indicated for women whose main concern is vaginal dryness during intercourse. Lubricants provide short-term relief from dryness and reduce dyspareunia. They can be water-based, which are water-soluble and have a tendency to dry out; oil based, which are more durable, but with a lower lubricating effect; or silicone-based. Some lubricants contain glycerin, propylene glycol, sweeteners and parabens, which may have an impact on the pH and osmolality of water-based products [29].

Vaginal moisturizers are insoluble hydrophilic cross-linked polymers with a characteristic bio-adhesiveness that is able to adhere to the epithelium of the vaginal wall by retaining water. They can also contain a large amount of excipients that influence the pH and the osmolality of the formulation. They can be used more regularly, rather than just in association with sexual activity, and have a longer lasting effect, improving the moisture of the vaginal mucosa and reducing the pH. The frequency of use is directly proportional to the severity of VVA [29]. The posology of the acute phase consists in local applications in the evening, before going to bed, for seven to ten consecutive days, so that they can act throughout the night, followed by two local applications per week to maintain the beneficial effects. The most commonly used moisturizers are based on hyaluronic acid (HA), a glycosaminoglycan produced by fibroblasts, which is the main component of the extracellular matrix. The possible action mechanism of hyaluronic acid is cell migration because it has a very high capacity to bind water, which may facilitate cellular movement [30]. Thus, in the case of tissue damage, HA may stimulate the migration and proliferation of fibroblasts and therefore the deposition of collagen fibers, in addition to stimulating neo-angiogenesis and re-epithelialization. If used on a regular basis, daily or every 2–3 days, HA based products improve symptoms of vaginal dryness, with an effect that has been compared with the effect of topical estrogen therapy [31]. Some adverse effects have been reported with the use of HA [32], but most have occurred after injections. They include local reactions namely bruising, erythema, swelling, and, rarely, more severe events such as tissue necrosis, infection, or pulmonary complications. To the best of our knowledge, no severe adverse effects have been reported with the use of HA-based vaginal moisturizers.

Other possible components of vaginal moisturizers are ozonides, intermediate products of ozone, which act as a biological reservoir preserving the therapeutic power of the molecule. In contact with biological tissue, ozonides activate quickly, stimulating the local microcirculation to induce neo-angiogenesis, promoting tissue repair, and inhibiting pro-inflammatory prostaglandins [33].

Oral vitamin D and vaginal vitamin E have been proposed for the treatment of VVA, but efficacy data are limited and sometimes discordant. Vitamin D stimulates the proliferation of the vaginal epithelium by activating the vitamin D receptor (VDR). Vaginal vitamin E is involved in the metabolism of all cells and prevents tissue damage caused by oxidants. This facilitates blood circulation, which consequently increases the metabolism of vaginal connective tissues and enhances the moisture and flexibility of vaginal walls [34,35,36].

Oral and vaginal probiotics for improving vaginal microbiota may be beneficial for the treatment of VVA symptoms, however, placebo-controlled trials that prove their effectiveness are lacking [37].

Oral phytoestrogens are not effective [38] while topical phytoestrogens seems to have a beneficial effect on VVA, improving genital symptoms, maturation index, vaginal pH, morphology, and expression of estrogen receptors in the vaginal epithelium [39], however, these are preliminary investigations that need to be verified in larger, prospective studies.

### 5.2. Hormonal Treatments

Hormone treatments of menopause (HTM), the association of estrogen-progestins, estrogen-bazedoxifene, tibolone, or exclusively estrogens in hysterectomized women, have a beneficial effect on many symptoms related to menopause including VVA. According to international guidelines, they are not recommended in women who suffer only from vaginal and vulvar symptoms, however, when they are used for primary indications, the evidence shows that HTM are able to restore the physiological vaginal pH, the maturation index, and the thickness of the vaginal epithelium, its vascularization and lubrication [40].

Tibolone is converted into metabolites that have tissue-specific agonistic estrogenic (3-alpha and 3-beta-hydroxytibolone) and progestogenic/androgenic (delta-4 tibolone) properties [41]. In post-menopausal women, tibolone normalizes the maturation index, alleviates atrophic vaginitis symptoms [42], and increases vaginal elasticity. Due to its androgenic activity, it has also been reported to have positive effects on sexual function [43].

The association of bazedoxifene with conjugated estrogens (BZA/CE), called tissue selective estrogen complex (TSEC), has also been reported to be effective in the treatment of moderate to severe VVA and its symptoms. At week 12, the BZA/CE combination increased superficial cells, decreased parabasal cells, decreased vaginal pH, and improved the most bothersome symptoms such as vaginal dryness or dyspareunia [39].

International guidelines recommend local hormonal therapy as a second step in the event of the ineffectiveness of vaginal lubricants and moisturizers [40]. Options available include estradiol, estriol, conjugated estrogens or promestriene gels, creams, ovules, tablets, or rings. These are specifically indicated for the treatment of VVA including dyspareunia. All estrogen-based vaginal products are more effective than a placebo for VVA. Vaginal estrogens are superior to lubricants and moisturizers in studies lasting at least six to twelve months [44,45].

The recommended dose is commonly a local daily application for two weeks as an attack therapy, and then application twice a week as maintenance therapy [30].

Estrogen absorption is limited to low doses, but it is not eliminated, especially in the early phases of treatment [45]. If the prescribed doses are taken, it is not necessary to associate a progestin for endometrial protection. Clinical evidence from large observational studies such as the Women’s Health Initiative Observational Study (WHI-OS) and the Nurse’s Health Study cohort did not find an increased risk of endometrial cancer in women who used vaginal estrogens [46]. However, observational studies have limitations and prospective, randomized controlled studies of long duration are lacking. The placement site inside the vagina is important as this has been suggested to affect the amount of estrogens reaching the endometrium [47]. The risks of stroke, breast cancer, pulmonary embolism, and deep vein thrombosis were not significantly different between vaginal estrogen users and non-users [46]. No significant differences in the safety profile of estradiol and estriol have been reported, while data on promestriene are scarce [48].

### 5.3. Selective Estrogen Receptor Modulator (SERM): Ospemifene

Ospemifene is the only selective estrogen receptor modulator (SERM) to be indicated for the treatment of VVA. It has been approved by the U.S. Food and Drug Administration (FDA) for the treatment of moderate to severe dyspareunia and by the European Medical Agency (EMA) for the treatment of moderate to severe VVA in women, with or without a uterus, who are not candidates for local estrogen therapy [49]. It exerts a positive effect on the vaginal epithelium while having, at the same time, a neutral or minimal effect on the other estrogen-dependent organs. In particular, it seems to have a neutral effect on the endometrium and the cardiovascular system, and an anti-estrogenic effect in pre-clinical studies on the breast. It is used at a dose of 60 mg daily.

The effects on the signs of VVA are visible after four weeks of treatment such as the increase of superficial cells, the reduction of basal cells, and the reduction of vaginal pH [50]. A significant effect on symptoms such as dryness and dyspareunia has been demonstrated to occur after 12 weeks of treatment [51]. Recently, the efficacy of ospemifene at a histological level in both vaginal and vulvar tissue has been demonstrated by observing increases in vaginal and vulvar epithelial thickness, glycogen content, proliferation index, and an increase in vaginal estrogen receptor alfa (ERα) [50,52]. Ospemifene has also been shown to improve atrophy of the vulvar vestibule and to normalize vestibular sensitivity by increasing the perception threshold at a vulvar level [53]. In a short-term study, it has also been shown to increase ratio type I and type III collagen at the vaginal level, suggesting possible beneficial long-term effects on vaginal connective tissue [52].

A current or previous thromboembolic event, vaginal bleeding of unknown origin, presence of signs or symptoms of endometrial hyperplasia, malignant tumor dependent on sex hormones, and ongoing breast carcinoma represent contra-indications. The EMA has also approved its administration in women with a previous breast cancer after the completion of treatment including adjuvant therapy and after performance of a control mammogram. The safety of this SERM on vaginal mucosa was first demonstrated in phase II and III clinical studies and has now been on the market for six years in the U.S. and for four years in some European countries including Italy. Ospemifene has an excellent safety profile that has been demonstrated by both randomized, double-blind, multicenter phase II and III placebo-controlled studies on a large number of patients, by the adverse event (AE) report and by the Post-Authorization Safety Study (PASS). The thromboembolic risk appears to be lower than with other SERMs. The incidence of cerebrovascular events was also lower in the cohort treated with ospemifene when compared to controls and in the cohort treated with other SERMs. Data on lipid and coagulative profiles were just as good, therefore the cardiovascular risk seems to be limited. Observed results regarding the risk of endometrial carcinoma meet the FDA criteria for endometrial safety. As with other compounds in this class, ospemifene seems to be safe on the breast in in vitro studies, in pre-clinical studies in animals, and in the surrogate parameters of breast safety [54,55,56].

### 5.4. Dehydroepiandrosterone (Prasterone)

Prasterone (dehydroepiandrosterone) has recently been introduced to the market for the treatment of VVA. It acts as a precursor of intracellular sex steroid androgens and estrogens. Since the conversion happens inside the cells, serum estradiol remains within the normal values for postmenopausal women, thereby probably avoiding the risk of systemic effects [57]. The efficacy of dehydroepiandrosterone (DHEA) has been demonstrated in a prospective, randomized, double-blind, placebo-controlled phase III clinical trial that examined the effects of daily intravaginal prasterone (6.5 mg) on four co–primary objectives, namely, the percentage of vaginal parabasal cells, percentage of vaginal superficial cells, vaginal pH, and moderate to severe dyspareunia, identified by women as the most bothersome VVA symptom. It may also be effective on the reduction of libido with a possible action on nerve endings, however, more scientific evidence is needed on this aspect [58].

Although data are limited to short-term studies on a relatively small number of patients, prasterone seems to be very safe. The endometrium is not affected by DHEA because the enzymes required to transform DHEA into estrogens are absent in the endometrium. Although no systemic increase of estrogen level has been reported, a history of breast cancer remains a contraindication.

### 5.5. Treatment Using Energy-Based Devices

#### 5.5.1. Laser Therapy

A new trend gaining in popularity in the treatment of VVA is the advent of energy-based devices. The most widely used are fractional microablative CO_2_ laser, non-ablative photothermal erbium: yttrium aluminum garnet (YAG) laser, and radiofrequency (RF)-based energy devices.

Laser or RF waves act by heating the connective tissue of the vaginal wall to 40 °C to 42 °C. In this way, they induce collagen contraction, neocollagenesis, vascularization, and growth factor infiltration that ultimately revitalizes and restores the elasticity and moisture of the vaginal mucosa. The proposed mechanism is the activation of heat shock proteins and tissue growth factors to stimulate new collagen synthesis and epithelial remodeling [59].

Recent reviews have suggested some potential benefits with the use of this technology in treating patients with VVA [60]. The efficacy of laser therapy in the treatment of VVA has been suggested by the improvement of GSM symptoms, VHI scores, and female sexual function index (FSFI) in many studies with its effectiveness at least as good as that of local estrogen based treatments [61,62]. However, none of these studies were sham or placebo controlled and the lack of sufficient information, especially concerning long-term safety, prompted the FDA in 2018 to warn against the indiscriminate marketing of laser treatments [63].

Although authors generally suggest that the procedure is well tolerated, being rapid and painless, increased vaginal pain, scarring, fibrosis, and vaginal wall lacerations have been reported [64].

The suggested treatment schedule involves three cycles at a distance of 30–40 days from each other as an attack therapy, with one cycle per year as maintenance therapy. Studies do not show how long the effects persist if the treatment is stopped and how often the treatment can be repeated.

#### 5.5.2. Radiofrequency Devices

Radiofrequency devices most commonly used by gynecologists are the transcutaneous temperature-controlled radiofrequency (TTCRF), and more recently, the low-energy dynamic quadripolar radiofrequency (DQRF). The mechanism of treatment is to trigger anatomical remodeling in the vaginal and vulvar tissues. There have been some small studies that prove its effectiveness on vaginal symptoms, sexual function as well as urinary symptoms, but again they have been small, non-randomized studies [65,66,67].

#### 5.5.3. Options for Treatment of Breast Cancer Survivors

In the case of women with previous or ongoing breast cancer, the options for treatment for VVA are unfortunately limited. All hormone-based therapies are contraindicated including vaginal isoflavone-based soy therapies, as there have been no studies on their safety in this cohort of women. Non-hormonal approaches are the first-line choices during or after breast cancer [68]. The options therefore are to offer these women moisturizers and vaginal lubricants, laser or radiofrequency treatments.

Another treatment that could be discussed with these women is ospemifene. Indeed, this SERM has been approved by the FDA for its use in women with previous breast cancer who have completed adjuvant therapy and have regular negative follow-ups. In cultured human breast tissue, ospemifene has been shown to induce a downregulation of ERα expression and decrease the proliferation of the cells, an effect that is consistent with the proposed anti-estrogenic activity of this SERM at the breast level [69]. In a recent small post-hoc analysis, a previous history of breast cancer did not appear to affect the efficacy or safety of ospemifene [54].

Finally, in this cohort of women, vaginal estrogen should be reserved for those patients who are unresponsive to non-hormonal remedies. The decision to use vaginal estrogen should be made in coordination with the woman’s oncologist. Importantly, an informed decision-making and consent process in which the woman is provided with the information and resources to consider the benefits and potential risks of vaginal estrogen administration should precede this decision [61]. Vaginally administered estrogen can be absorbed in small amounts without raising blood levels, however, it may potentially stimulate occult breast cancer and could interfere with tamoxifen or aromatase inhibitors (AI) [70]. DHEA seems to be safe on breast tissue as it maintains serum estradiol within normal post-menopausal values, thus avoiding the risk of systemic effects. However, there have been no studies in a cohort of breast cancer survivors.

## 6. Conclusions

VVA is still an under-addressed, under-diagnosed, and consequently under-treated condition. It affects the quality of life of millions of postmenopausal women. Although many treatment approaches have been developed over the past decade, many still lack proper efficacy data and, more importantly, safety data are still insufficient. More safety data are also needed for the treatment of this condition in hormone-dependent breast cancer patients. Ospemifene, DHEA, and laser treatments seem to be promising alternatives for patients who cannot use hormones; however, their safety needs further research.

## Appendix A

**Table A1:** Prevalence of vulvovaginal atrophy according to the most recent surveys and studies.

Study	Author	Year	Women’s Age Range	Method of Study	Prevalence
“Women’s voices in the menopause” survey	Nappi et al. [7]	2010	55–65 years	Computer-assisted web interviews	39%
VIVA survey	Nappi et al. [8]	2012	55–65 years	Online survey	45%
AGATA study	Palma et al. [9]	2016	59 years (average)	Interview and gynecological examination	79%
The Women’s EMPOWER Survey	Kingsberg et al. [10]	2017	45–90 years	Online survey	39–51%
EVES study	Palacios et al. [5]	2018	45–75 years	Questionnaires and gynecological examination	90%
GENISSE study	Moral et al. [12]	2018	30–75 years	Interview and gynecological examination	70%
ANGEL study	Cagnacci et al. [13]	2019	40–55 years	Interview and gynecological examination	36.8%
